# Basilar Artery Occlusion Secondary to Cocaine-Associated Cardiomyopathy: A Case of Cardioembolic Stroke With Poor Outcome Despite Successful Reperfusion

**DOI:** 10.7759/cureus.107165

**Published:** 2026-04-16

**Authors:** Alireza Izadian Bidgoli, Alberto Gomez Veliz, Atharv Joshi, Leah M Akl, Allen Rubinchuk

**Affiliations:** 1 Internal Medicine, American University of the Caribbean School of Medicine, Cupecoy, SXM; 2 Internal Medicine, Jackson Memorial Hospital, Miami, USA; 3 Research, Orlando College of Osteopathic Medicine, Winter Garden, USA

**Keywords:** basilar artery occlusion, cardioembolic stroke, endovascular thrombectomy, multisystem complications, posterior circulation stroke

## Abstract

Basilar artery occlusion (BAO) is a neurologic emergency associated with high morbidity and mortality, in which successful reperfusion does not consistently translate into favorable functional outcomes. While futile recanalization has been well described, the underlying mechanisms contributing to poor outcomes remain incompletely understood. We present the case of a 54-year-old male with polysubstance use who presented with altered mental status and focal neurologic deficit, and was found to have BAO. He underwent emergent endovascular thrombectomy with complete reperfusion (thrombolysis in cerebral infarction (TICI) grade 3). Despite this, post-procedural imaging demonstrated extensive multifocal infarctions involving the posterior circulation and supratentorial territories. Further evaluation revealed newly diagnosed severe systolic dysfunction with an ejection fraction of 20%, raising concern for a cardioembolic source. In the context of confirmed cocaine use, this finding suggests cocaine-associated cardiomyopathy as a potential underlying mechanism contributing to thrombus formation and embolization. The pattern of widespread infarction across multiple vascular territories supports a systemic embolic process rather than isolated large vessel occlusion. Despite hemodynamic stability and appropriate medical management, the patient remained significantly functionally impaired. This case highlights cocaine-associated cardiomyopathy as a likely underrecognized and clinically important cardioembolic mechanism in posterior circulation stroke. It underscores the importance of early cardiac evaluation in patients with multifocal infarction patterns and substance use, and demonstrates how severe systolic dysfunction may contribute to poor neurologic outcomes despite technically successful reperfusion.

## Introduction

Posterior circulation stroke represents a clinically distinct subset of ischemic stroke, associated with high morbidity and mortality despite advances in diagnosis and treatment [1]. Among these, basilar artery occlusion (BAO) is one of the most severe cerebrovascular emergencies, with mortality rates approaching 40-80% without timely reperfusion [1]. Although advances in endovascular therapy (EVT) have significantly improved rates of recanalization and clinical outcomes, a substantial proportion of patients continue to experience poor neurologic recovery despite technically successful reperfusion [2,3].

The variability in outcomes following EVT highlights the importance of underlying stroke mechanisms. Cardioembolism is a major contributor to ischemic stroke, particularly in patients with cardiac dysfunction, where impaired ventricular function, blood stasis, and endothelial activation promote thrombus formation and embolization [4,5]. In addition, cocaine use has been associated with both vascular and cardiac pathology, including vasospasm, endothelial injury, prothrombotic effects, and cardiomyopathy, all of which may increase the risk of ischemic stroke [6]. Despite these associations, cocaine-associated cardiomyopathy as a cardioembolic source in posterior circulation stroke remains underrecognized.

Here, we present a case of BAO with extensive multifocal infarction involving both posterior and supratentorial territories in the setting of newly diagnosed severe systolic dysfunction and polysubstance use. This case highlights cocaine-associated cardiomyopathy as a potential cardioembolic mechanism and underscores the importance of a mechanism-based approach to stroke evaluation in patients with complex clinical presentations.

## Case presentation

Clinical presentation 

A 54-year-old male with an unknown past medical history presented on March 11, 2026, with altered mental status, left-sided weakness, and left gaze deviation. Initial evaluation revealed a severe neurologic deficit concerning a posterior circulation stroke. The patient was not a candidate for intravenous thrombolysis due to an unknown last known well time. Neuroimaging demonstrated a BAO, and the patient underwent emergent endovascular thrombectomy (EVT) with successful reperfusion (thrombolysis in cerebral infarction (TICI) grade 3). Following the procedure, he was admitted to the neurocritical care unit for further management. 

Evaluation (history, examination, laboratory findings, and imaging)

On neurologic examination, the patient was nonverbal and intermittently moaning, with limited ability to participate in the exam. He followed simple commands inconsistently. Cranial nerve examination demonstrated pupils equal and reactive to light. Extraocular movements were limited but notable for left gaze deviation. Facial symmetry appeared grossly intact without clear asymmetry. Assessment of lower cranial nerves, including gag reflex and palate elevation, was limited due to the patient’s mental status. No obvious tongue deviation was observed. Overall, the cranial nerve examination was limited by poor participation. Motor examination revealed antigravity strength in the left upper and lower extremities. The right lower extremity demonstrated brief antigravity movement with rapid drift, while the right upper extremity exhibited no voluntary movement, consistent with a dense right-sided hemiparesis. Sensation was intact to light touch.

**Figure 1:**
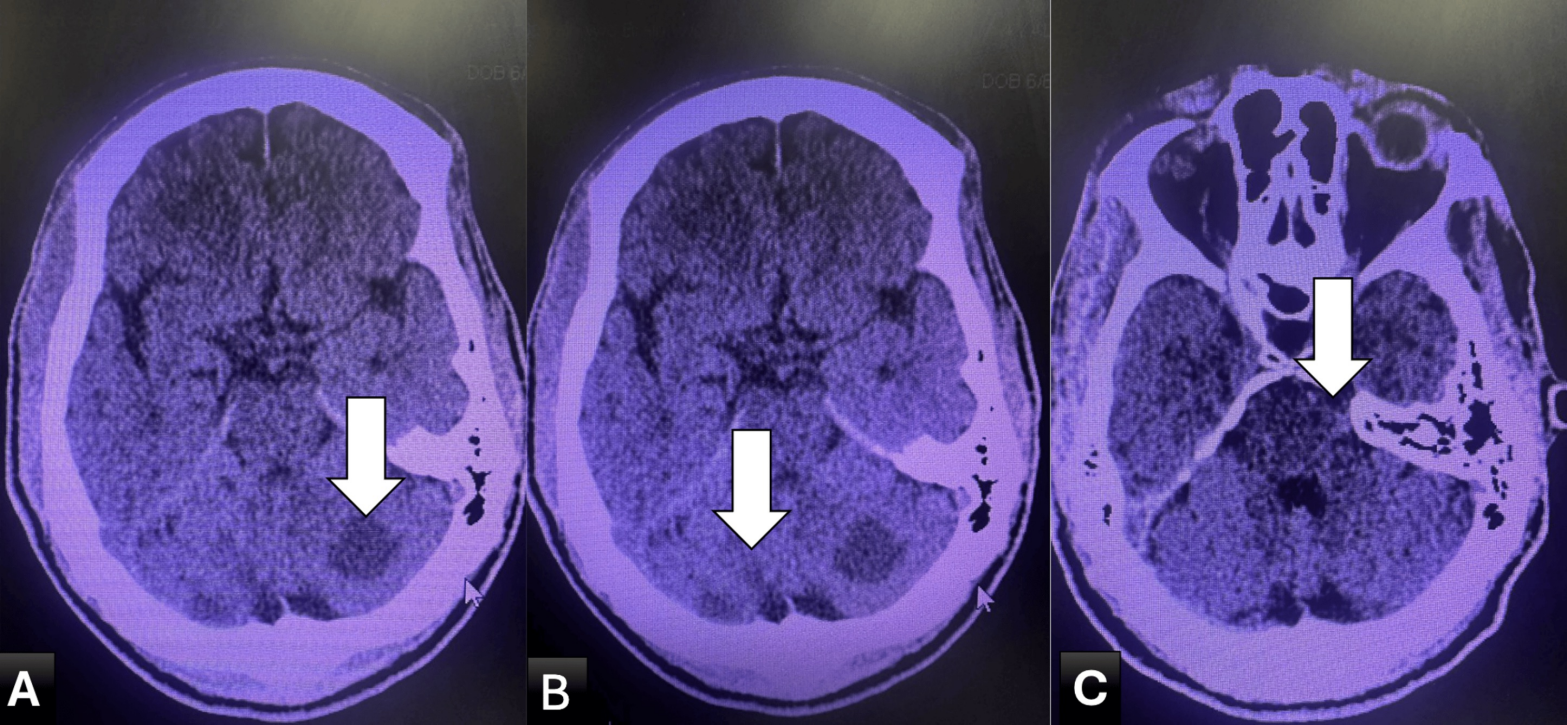
Non-contrast CT of the head demonstrating posterior circulation ischemia. (A-C) Axial CT images showing hypodensities (arrows) involving the bilateral cerebellar hemispheres (A, B) and the brainstem (C), consistent with early posterior circulation infarction. CT, computed tomography.

**Figure 2:**
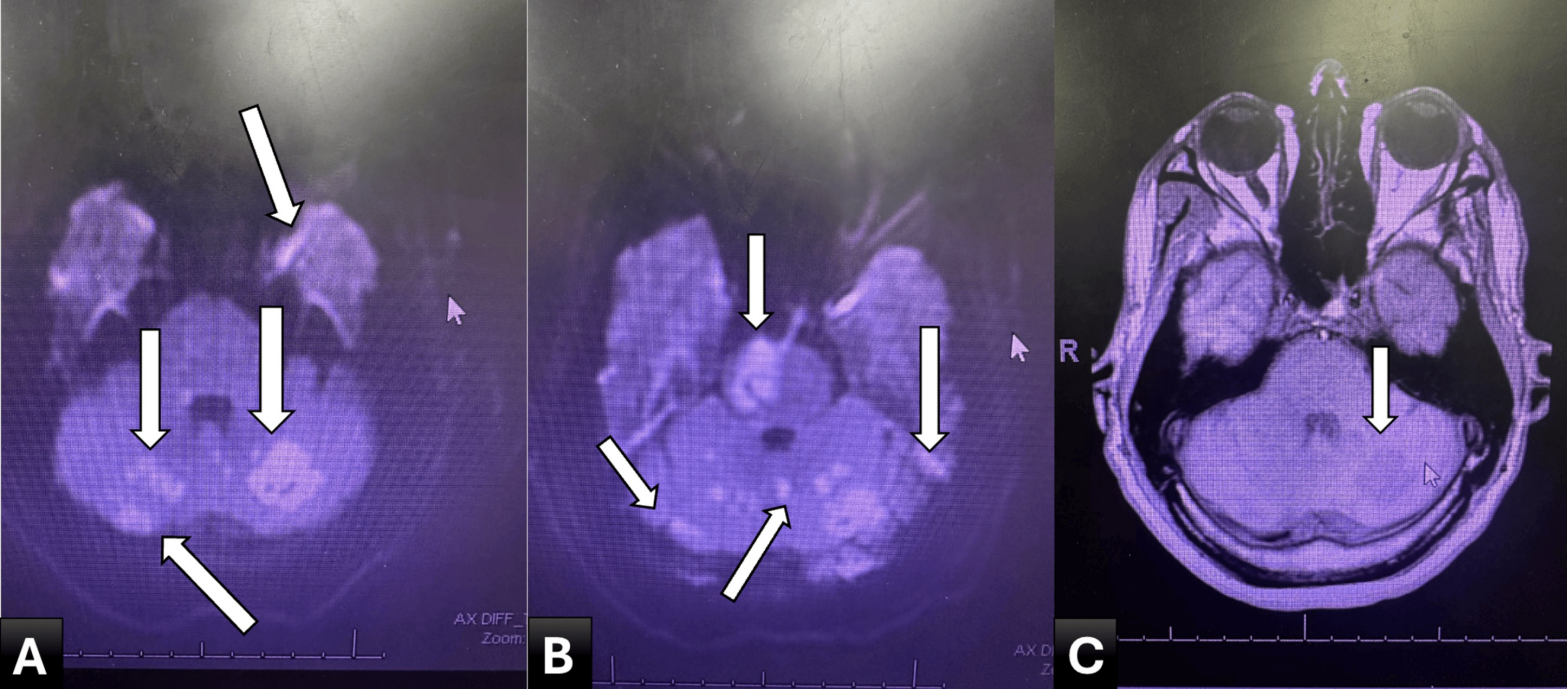
MRI of the brain. (A, B) DWI sequences showing areas of restricted diffusion within the bilateral cerebellar hemispheres and brainstem (arrows), consistent with acute infarction. (C) Corresponding axial sequence demonstrating posterior circulation involvement. MRI, magnetic resonance imaging; DWI, diffusion-weighted imaging.

Initial non-contrast computed tomography (CT) of the head demonstrated early ischemic changes involving the posterior circulation, including the cerebellum and brainstem (Figure 1). Subsequent magnetic resonance imaging (MRI) of the brain confirmed multifocal acute infarctions involving the bilateral cerebellar hemispheres, occipital lobes, thalamus, and brainstem (Figure 2).

Laboratory evaluation was performed as part of the stroke workup, with key findings summarized in Table 1. Transthoracic echocardiography revealed newly identified severe systolic dysfunction with an ejection fraction of 20%, raising concern for a cardioembolic source. The etiology was therefore considered an embolic stroke of undetermined source (ESUS) versus artery-to-artery embolism in the setting of suspected vertebral artery dissection.

**Table 1:** Summary of the patient’s initial laboratory evaluation on presentation. The findings demonstrate microcytic anemia and leukocytosis without evidence of hemolysis. MCV, mean corpuscular volume; LDH, lactate dehydrogenase.

Parameter	Value	Units	Reference Range
Hemoglobin	7.8	g/dL	12-16
Hematocrit	27.2	%	36-46
MCV	62.2	fL	80-100
WBC count	24.1	×10³/µL	4.0-11.0
Platelet count	308	×10³/µL	150-400
Sodium	136	mmol/L	135-145
Potassium	4.0	mmol/L	3.5-5.0
Bicarbonate (CO₂)	20	mmol/L	22-28
Creatinine	0.60	mg/dL	0.6-1.3
Calcium	8.2	mg/dL	8.6-10.2
Total bilirubin	0.4	mg/dL	0.1-1.2
LDH	251	U/L	140-280
Haptoglobin	208	mg/dL	30-200

During hospitalization, the patient developed dysphagia with delayed swallow initiation and aspiration, confirmed on a modified barium swallow study demonstrating laryngeal penetration and tracheal aspiration. Laboratory studies during admission were notable for stable renal function. Toxicology screening was positive for cocaine and cannabis, suggesting recent polysubstance use, which may have contributed to the patient’s stroke through mechanisms such as vasospasm, endothelial injury, and prothrombotic effects. Microbiological evaluation of respiratory specimens demonstrated heavy growth of *Haemophilus influenzae* (beta-lactamase negative) with moderate respiratory flora, consistent with aspiration pneumonia.

Management****

Following successful thrombectomy, the patient was managed with aspirin 81 mg daily and high-intensity statin therapy (rosuvastatin 20 mg daily) for secondary stroke prevention. Blood pressure parameters were maintained within target range (systolic blood pressure 100-180 mmHg).

Given the newly diagnosed heart failure with reduced ejection fraction (HFrEF, EF 20%), guideline-directed medical therapy was initiated, including metoprolol tartrate and dapagliflozin, with plans to escalate therapy to include sacubitril/valsartan and spironolactone as clinically tolerated.

His hospital course was complicated by acute metabolic encephalopathy and delirium, likely multifactorial in the setting of acute stroke, hospitalization, and substance use. Although toxicology screening was positive for cocaine and cannabis, there was no clear clinical evidence of withdrawal symptoms contributing to his presentation. Psychiatry was consulted, and he was managed with quetiapine and as-needed olanzapine, along with standard delirium precautions.

Due to persistent dysphagia and high aspiration risk, the patient was placed on nil per os (NPO) status and initiated on total parenteral nutrition (TPN). A percutaneous endoscopic gastrostomy (PEG) tube placement was planned following appropriate peri-procedural management, including temporary cessation of antiplatelet therapy.

For aspiration pneumonia, the patient was treated with piperacillin-tazobactam, with clinical improvement.

The patient remained hemodynamically stable and demonstrated gradual neurologic improvement, allowing transfer from the neurointensive care unit to a step-down unit.

Clinical outcome

At the time of the last evaluation, the patient remained nonverbal but was able to follow simple commands, with persistent dense right hemiplegia and significant functional impairment. Dysphagia initially persisted following the acute event, necessitating strict aspiration precautions and enteral nutritional support. A PEG tube was subsequently placed without immediate complication, and the patient tolerated enteral feeding well, with adequate nutritional support and no evidence of active bleeding or tube-related complications.

Follow-up speech and swallowing evaluation demonstrated no evidence of overt aspiration; however, given persistent neurologic deficits, continued speech therapy was recommended to optimize functional recovery. The patient remained hemodynamically and medically stable throughout the latter portion of hospitalization. Disposition planning was complicated by social factors, including polysubstance use and housing instability, ultimately requiring multidisciplinary coordination. The patient was planned for transfer to a skilled nursing facility for ongoing rehabilitation and long-term care.

## Discussion

Pathophysiology and mechanisms of stroke in this case

This case demonstrates a likely cardioembolic BAO in the setting of newly identified severe systolic dysfunction and polysubstance use, with cocaine as a potential contributor to cardiomyopathy and embolic risk [4-6]. Despite successful reperfusion with EVT (TICI 3), the patient developed extensive multifocal infarctions involving both posterior and supratentorial territories, a pattern more consistent with a systemic embolic process than with an isolated large-vessel occlusion alone [2-4]. Posterior circulation strokes account for a minority of ischemic strokes but are associated with disproportionately high morbidity and mortality because they involve critical structures, including the brainstem, cerebellum, and occipital lobes [1,7-9]. Among these, BAO is one of the most severe cerebrovascular emergencies and is associated with a high risk of disability and death without timely reperfusion [1,8,9].

Although EVT has significantly improved recanalization rates and clinical outcomes in BAO, a substantial proportion of patients continue to experience poor neurologic recovery despite technically successful reperfusion [1-3]. Prior literature has shown that outcomes after BAO are influenced not only by recanalization status but also by infarct burden, stroke severity, time to reperfusion, and underlying stroke mechanism [1-3]. In this case, these factors are most consistent with a systemic embolic process, likely driven by underlying severe systolic dysfunction in the setting of cocaine use, resulting in extensive multifocal infarction and poor neurologic recovery. Cardioembolism is a major mechanism of ischemic stroke, particularly in patients with underlying cardiac dysfunction [4,5,10]. In heart failure with reduced ejection fraction, impaired ventricular contractility promotes blood stasis, endothelial activation, and a prothrombotic state, thereby increasing the risk of intracardiac thrombus formation and cerebral embolization [5]. Cardiac emboli may fragment and distribute across multiple vascular territories, producing multifocal infarction patterns similar to those seen in this patient [4,10].

Cocaine use may further amplify stroke risk through both vascular and cardiac mechanisms [6]. Cocaine has been associated with vasospasm, endothelial injury, and prothrombotic effects, all of which increase the likelihood of ischemic stroke [6]. In addition, cocaine has been linked to myocardial injury and cardiomyopathy, providing a plausible mechanism by which substance use may contribute indirectly to cardioembolic stroke [6]. In this patient, the combination of severe systolic dysfunction (ejection fraction 20%) and recent cocaine use supports cocaine-associated cardiomyopathy as a plausible contributor to thrombus formation and subsequent embolization [5,6]. The presence of infarctions involving both posterior and supratentorial territories further supports a systemic embolic source rather than isolated basilar thrombosis alone [4]. This distinction is clinically important because it shifts the interpretation of the event from a purely focal vascular occlusion to a broader systemic and cardiac process driving stroke pathogenesis [4,5]. Despite complete recanalization, the extensive embolic burden and widespread infarction likely contributed to the patient’s poor neurologic outcome [2,3,11].

Comparative analysis of this case with the current literature 

Clinical Presentation

BAO is a rare but severe form of ischemic stroke, accounting for approximately 1% of all strokes and associated with high morbidity and mortality [1,8,9]. Patients with BAO commonly present with decreased level of consciousness, cranial nerve deficits, gaze abnormalities, and motor impairment, findings that are consistent with this patient’s presentation of altered mental status, gaze deviation, and focal neurologic deficits [7-9]. However, unlike more typical BAO presentations, this patient demonstrated extensive multifocal infarctions involving both posterior circulation and supratentorial territories, a pattern more suggestive of an embolic process affecting multiple vascular distributions than of isolated basilar thrombosis alone [4,10]. These imaging findings make the case clinically distinctive and support a broader etiologic evaluation beyond the occluded basilar artery itself [4,12].

Diagnostic Workup

Magnetic resonance imaging in BAO typically demonstrates infarction involving structures supplied by the vertebrobasilar circulation, including the brainstem, cerebellum, thalami, and occipital lobes [7,8]. In our patient, this expected posterior circulation pattern was present, but the additional supratentorial infarcts raised concern for a systemic embolic source rather than a single focal arterial event [4,10]. Current literature emphasizes the importance of evaluating ESUS through careful cardiac assessment, as occult cardiac dysfunction may be identified even in patients without a known prior cardiac history [4,5,12]. In this case, newly identified severe systolic dysfunction with an ejection fraction of 20% provides a plausible substrate for intracardiac thrombus formation and subsequent embolization [5]. Cocaine use introduces an additional mechanistically relevant factor, as it has been associated with vasospasm, endothelial injury, thrombotic risk, myocardial injury, and cardiomyopathy, all of which may contribute to ischemic stroke through both vascular and cardiac pathways [6]. Together, these findings support the possibility that cocaine-associated cardiomyopathy contributed to a cardioembolic mechanism in this patient [5,6].

Management

Endovascular thrombectomy has become an important treatment modality for acute BAO, with randomized trials and reviews demonstrating improved reperfusion rates and better functional outcomes in selected patients when compared with medical therapy alone [1-3,9,13]. In this case, successful reperfusion was achieved with thrombectomy, with a final TICI 3 result documented in the manuscript. However, prior studies have shown that technically successful recanalization does not necessarily translate into favorable neurologic recovery, particularly in patients with extensive infarction, delayed presentation, or complex underlying stroke mechanisms [1-3,11]. Secondary prevention and supportive management in this case, including antiplatelet therapy, high-intensity statin therapy, and initiation of guideline-directed medical therapy for heart failure, are consistent with standard management principles for ischemic stroke and newly recognized systolic dysfunction [5,14].

Outcome

BAO remains associated with substantial mortality and long-term disability despite advances in reperfusion therapy [1,8,9]. This is consistent with our patient’s persistent neurologic deficits, including nonverbal status, dense hemiplegia, dysphagia, and significant functional impairment at the time of last evaluation. Dysphagia and aspiration pneumonia are well-recognized complications after severe stroke and contribute significantly to post-stroke morbidity [15]. In this case, those complications likely increased the overall illness burden, although the patient’s poor neurologic outcome was more fundamentally related to the severity and distribution of infarction at presentation [2,3,11,15].

Literature Summary and Key Distinction

Recent literature on BAO emphasizes early recognition, timely reperfusion, and comprehensive evaluation of stroke etiology as major determinants of outcome [1-4,9]. This case is consistent with prior observations that embolic mechanisms, especially in the setting of cardiac dysfunction, can produce larger and more multifocal infarction patterns than are typically seen with isolated local thrombosis [4,5,10]. However, the distinguishing feature of this case is the likely contribution of newly identified severe systolic dysfunction in the setting of cocaine use as a plausible cardioembolic source [5,6]. While poor functional outcome despite successful reperfusion and the occurrence of hospital-related complications are already well described in the literature [2,11,15], this case highlights cocaine-associated cardiomyopathy as a likely underrecognized and clinically important mechanism that may contribute to posterior circulation stroke [16]. The combination of severe systolic dysfunction, active cocaine use, and multifocal infarction across multiple vascular territories supports a systemic embolic process and underscores the importance of a mechanism-based approach to stroke evaluation in complex BAO presentations [4-6]. 

What we learned from this case 

This case highlights cocaine-associated cardiomyopathy as a likely underrecognized and clinically important cardioembolic mechanism in posterior circulation stroke. In patients presenting with BAO and newly identified severe systolic dysfunction, particularly in the setting of polysubstance use, cardiac dysfunction should be strongly considered as a primary driver of embolic risk.

The presence of multifocal infarctions involving both posterior and supratentorial territories in this patient supports a systemic embolic mechanism rather than an isolated large-vessel occlusion. Recognition of this radiographic pattern should prompt early and comprehensive cardiac evaluation, even in the absence of a previously established cardiac history. In this case, severe left ventricular dysfunction likely created a prothrombotic environment conducive to intracardiac thrombus formation and subsequent embolization, with cocaine use serving as a potential precipitating factor through its known cardiotoxic effects.

This case also demonstrates that successful reperfusion in BAO does not guarantee favorable neurologic outcomes when extensive embolic burden and widespread infarction are present. Rather than reflecting procedural failure, poor recovery in such cases may be better explained by the underlying embolic mechanism and infarct distribution at presentation.

Overall, this case underscores the importance of identifying the underlying embolic source in complex stroke presentations. Early recognition of cocaine-associated cardiomyopathy has important implications for diagnostic evaluation, risk stratification, and secondary prevention in patients with multifocal infarction patterns and substance use.

## Conclusions

BAO remains a devastating form of posterior circulation stroke in which successful reperfusion does not guarantee favorable neurologic outcomes. This case highlights the importance of underlying stroke mechanisms, demonstrating how newly identified severe systolic dysfunction in the setting of cocaine use may serve as a plausible cardioembolic source. The observed infarction pattern is consistent with a systemic embolic process rather than an isolated vascular occlusion.

While poor outcomes following successful recanalization are well described, this case emphasizes the need to move beyond procedural success and focus on identifying the underlying embolic source. In patients with multifocal infarction and substance use, early recognition of cardiac dysfunction is critical for accurate diagnosis, risk stratification, and secondary prevention.

Overall, this case underscores cocaine-associated cardiomyopathy as a likely underrecognized and clinically important contributor to posterior circulation stroke and highlights the importance of integrating cardiac and substance-related factors into the evaluation of complex stroke presentations.
